# The Surprising Dynamics of Electrochemical Coupling at Membrane Sandwiches in Plants

**DOI:** 10.3390/plants12010204

**Published:** 2023-01-03

**Authors:** Ingo Dreyer, Fernando Vergara-Valladares, Franko Mérida-Quesada, María Eugenia Rubio-Meléndez, Naomí Hernández-Rojas, Janin Riedelsberger, Sadith Zobeida Astola-Mariscal, Charlotte Heitmüller, Mónica Yanez-Chávez, Oscar Arrey-Salas, Alex San Martín-Davison, Carlos Navarro-Retamal, Erwan Michard

**Affiliations:** 1 Electrical Signaling in Plants (ESP) Laboratory, Centro de Bioinformática, Simulación y Modelado (CBSM), Facultad de Ingeniería, Universidad de Talca, 2 Norte 685, Talca 3460000, Chile; 2 Doctorado en Ciencias mención Modelado de Sistemas Químicos y Biológicos, Universidad de Talca, 2 Norte 685, Talca 3460000, Chile; 3 Department of Plant Biotechnology, Institute of Plant Genetics, Leibniz Universität Hannover, 30419 Hannover, Germany; 4 Instituto de Ciencias Biológicas, Universidad de Talca, Campus Talca, Avenida Lircay, Talca 3460000, Chile; 5 Instituto de Investigación Interdisciplinaria, Universidad de Talca, 2 Norte 685, Talca 3460000, Chile; 6 Cell Biology and Molecular Genetics, University of Maryland, College Park, MD 20742–5815, USA

**Keywords:** computational cell biology, modelling, nutrient transport, plant biophysics, mathematical model, plant–fungus interaction

## Abstract

Transport processes across membranes play central roles in any biological system. They are essential for homeostasis, cell nutrition, and signaling. Fluxes across membranes are governed by fundamental thermodynamic rules and are influenced by electrical potentials and concentration gradients. Transmembrane transport processes have been largely studied on single membranes. However, several important cellular or subcellular structures consist of two closely spaced membranes that form a membrane sandwich. Such a dual membrane structure results in remarkable properties for the transport processes that are not present in isolated membranes. At the core of membrane sandwich properties, a small intermembrane volume is responsible for efficient coupling between the transport systems at the two otherwise independent membranes. Here, we present the physicochemical principles of transport coupling at two adjacent membranes and illustrate this concept with three examples. In the supplementary material, we provide animated PowerPoint presentations that visualize the relationships. They could be used for teaching purposes, as has already been completed successfully at the University of Talca.

## 1. Introduction

Transport processes across cellular membranes are essential components of cell biology, as they are involved in most cellular functions such as energy supply, water fluxes, ion homeostasis, protein maturation, and cell signaling. In plants, where there is no contractile organ equivalent to the heart, the motor that dispatches water and nutrients in the organism is constituted by the activity of proton ATPases and ion transporters at the membranes. In concerted actions, they drive the bulk flow of xylem sap from the soil solution to the leaves and of elaborated sap from photosynthetic source organs to heterotrophic sinks. A detailed mechanistic description of membrane transporters is mandatory to understand their functions in plant nutrition, plant cell homeostasis, and associated plant growth. It also requires a description of the biophysical contexts in which these transporters operate. Of particular importance in this regard is the cell-specific organization of the membranes in which the transporters are localized, with their subtleties and remarkable structures that confer the transport system its specificity.

Transmembrane transport processes are very often studied by simplifying the experimental system to a single membrane. While this simplification is certainly pertinent in a variety of studies, some plant cell systems exhibit remarkable features that deserve special attention. These structures can be characterized as membrane sandwiches, consisting of a small volume compartment between two membranes. Such a dual-membrane system is the rule rather than the exception in the plant. The zone between two independent membrane systems can be apoplastic, when two non-connected plasma membranes face each other, or cytosolic, when one is an organelle membrane. Apoplastic membrane sandwiches are located at the interface of cells that are not connected by plasmodesmata; for instance, along the phloem with the sieve element/companion cell (SE/CC) complex, at guard cells, and in pollen tubes growing in the style. Highly relevant from an agronomical point of view, membrane sandwiches are also found at the interface between plant cells and cells of a foreign intruding organism, be it a parasite or a symbiont.

Despite the omnipresence of membrane sandwiches in plants, their dynamic properties have hardly been explored, a fact surprising enough considering their main importance in plant development. Only recently have we begun to systematically investigate the peculiar dynamics of membrane sandwiches using computational cell biology approaches (for instance, I. Dreyer, the 18th International Workshop on Plant Membrane Biology 2019, Glasgow, Abstract P1.89). In this article, we summarize some of the findings on this topic and place them in a physiological context. Although the two adjacent membranes of a sandwich are electrically isolated from each other, the transport across one of the membranes is strongly coupled to the transport processes across the other, enabling rapid and cost-effective transport mechanisms for efficient, coordinated ion, and nutrient fluxes.

## 2. Fundamental Biophysical Properties of Membrane Sandwiches in Plants

As stated above, a membrane sandwich consists of two adjacent membranes separated by a relatively small distance. The first examples that come to mind when thinking of such a structure are certainly the outer and inner membrane of mitochondria, which separate the 6–8 nm wide intermembrane space [1], or the outer and inner membranes of chloroplasts, with an intermembrane space of about 10–20 nm thick [2], and the thylakoid membrane as the third membrane in series, or the two membranes of the nuclear envelope that are separated by 30–50 nm [3]. Additionally, extensions of organelles, called stromules, would be an example [4]. However, in this article, we do not refer to these examples of very thin intermembrane spaces where electrostatic interfacial phenomena, such as Guy–Chapman layers, play a dominant role. Instead, we refer to structures with larger intermembrane spaces, as they are omnipresent in plants. Transmission electron microscopy pictures may allow us to conclude that the usual distance between the plasma membranes of two neighboring cells is about 100–1000 nm [5,6,7].

To illustrate the effects in these structures, we considered, as an example, a 1 μm^2^ membrane patch and a distance between the two membranes of 100 nm, as determined for the peri-arbuscular space in arbuscular mycorrhizal symbiosis [8]. The membrane interface compartment of this patch has a volume of 0.1 fL (=10^−16^ L). With such a small volume, substrate concentrations in the intermediate space can be subject to significant fluctuations very quickly. For instance, the transport of just 60 molecules/ions per μm^2^ into or out of this interface changes the concentration by 1 μM (Δc = *N* × *N_A_*^−1^ × *Vol*^−1^; with the Avogadro constant *N_A_*, the number of molecules *N,* and the volume *Vol*). Taking into account that plant membrane transporters catalyze the movement of 0.5 × 10^3^ to >10^6^ molecules/s and considering that they are expressed at densities of 1 to 10 transporters/μm^2^ [9,10,11], abrupt concentration changes in the millimolar range theoretically take from milliseconds to a few seconds, which would represent one of the fastest physiological processes in plants. With a bigger cleft, the volume is correspondingly larger and the buffer capacity of the intermembrane space is proportionally greater. However, even with a distance of 1000 nm, the membrane interface compartment would still have a volume of not more than 1 × 10^−15^ L per μm^2^ patch. In this case, the movement of ~6 × 10^5^ ions per μm^2^ across one membrane, which can be mediated by an ion channel in less than a second (~100 fA), would change the concentration in the intermembrane space by 1 mM. Thus, even in a general plant physiological context, membrane sandwich effects may not be negligible.

**Figure 1:**
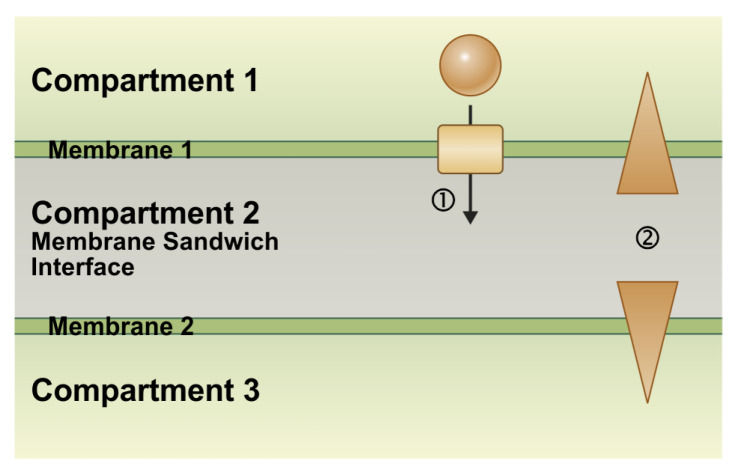
The basic principle of coupling transport processes at membrane sandwiches. Two adjacent membranes separate three compartments. In one possible scenario, compartments 1 and 3 are the cytosol of two neighboring cells, the membranes are the plasma membranes, and compartment 2 is the apoplast. In another scenario, compartment 1 is the apoplast, compartment 2 the cytosol, and compartment 3 is an intracellular organelle, such as the vacuole. In this case, membrane 1 is the plasma membrane and membrane 2 is the organelle membrane (e.g., the tonoplast). (1) The transport of a molecule across membrane 1 changes the concentrations of that substance in compartments 1 and 2. (2) The altered concentrations, in turn, change the transmembrane concentration gradients not only at membrane 1 but also at membrane 2, thereby affecting the transport processes even across that membrane.

Transport processes at the two separate membranes are ruled by the respective transmembrane electrical and chemical gradients. Since the sandwich membranes face the common intermembrane space, the rapidly adjusting concentrations in the tiny volume affect, therefore, the chemical gradients across both membranes. Thus, when transporter activity in one membrane alters the intermembrane concentration, it directly affects the chemical gradient across the second membrane, thereby coupling transport across one membrane with transport across the other, even though the two are not connected (Figure 1). This coupling has fundamental implications for transport mechanisms at sandwich membranes, which have different properties than those known from isolated membranes. In the following, we present the computational cell biological simulation of three examples of transport across sandwich membranes. To facilitate the understanding, we visualize the transport processes and their driving forces in animations that can be found in the Supplementary Materials.

## 3. Example 1: Remote Control of Phloem (Re-)Loading

Long-distance transport in the phloem of vascular plants bridges the distance between the green tissues where photosynthesis occurs and heterotrophic organs, such as roots, flowers, and fruits, where the products of photosynthesis are used for growth and/or storage. Via the phloem vasculature, various molecules synthesized in the leaves (e.g., sugars, amino acids, and other nitrogenous or phosphorus-containing compounds) are transported alongside inorganic ions from the source tissues, which produce more assimilates than they need, to the consuming sink tissues [12]. The transport phloem between source and sink tissues is not a passive transport tube, but a living tissue that also contributes significantly to the maintenance and growth of the plant axis. During the passage from the source to the sink along the transport phloem, there is a dynamic release and retrieval of photoassimilates and ions between the phloem tissue and the surrounding parenchyma cells [13]. Usually, the underlying membrane transport processes are energized by ATP-consuming proton pumps. However, retrieval can also be energized by a potassium battery, i.e., the K^+^ gradient between phloem cytosol and the apoplast [14,15,16]. Voltage-gated K^+^ channels of the AKT2-type [17,18,19,20,21,22] play a unique role in this process.

**Figure 2:**
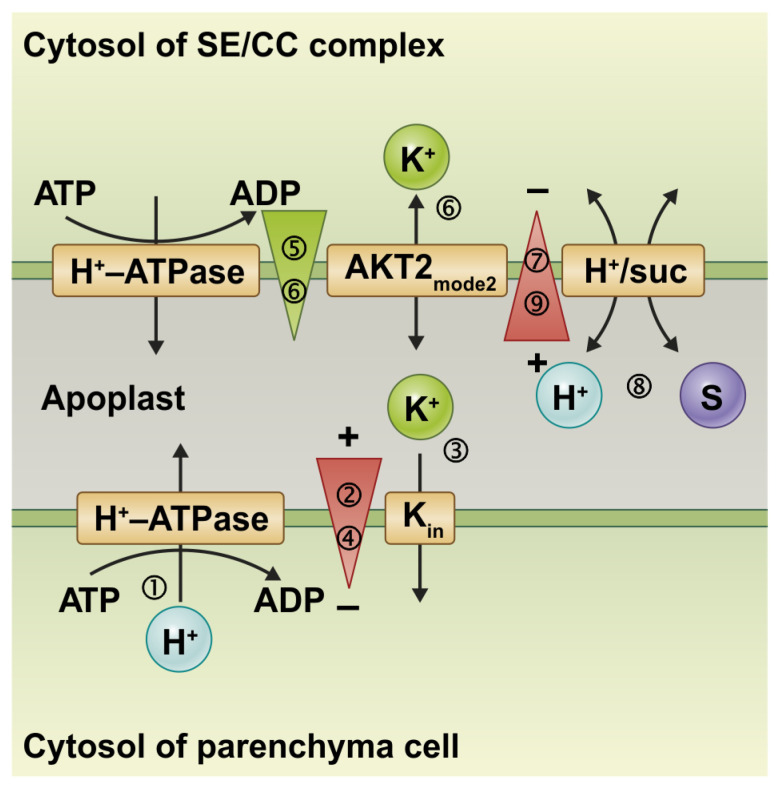
Electrochemical coupling allows remote control during phloem re-loading processes. See text for details. An animated version of this Figure can be found in the Supplementary Materials (Animation S1).

Interestingly, the associated transport processes couple in a way that the surrounding parenchyma cells can remotely control the reloading of photoassimilates into the phloem [14] (Figure 2; Animation S1). To illustrate this phenomenon, the different transport processes are presented step by step. Starting from the global steady state of the system, the H^+^-ATPase of the parenchyma cell pumps a proton out of the cell (1), which leaves the membrane voltage more negative inside (2). This additional electrical gradient drives the influx of a K^+^ ion into the parenchymal cell via a K^+^ uptake channel (3), which in turn relieves the additional electrical gradient (4) but locally causes an additional K^+^ concentration gradient across the phloem membrane (5). This additional [K^+^] gradient drives the efflux of a K^+^ ion via AKT2-type channels from the phloem into the apoplast, offsetting the additional [K^+^] gradient (6). The transmembrane charge transport creates an additional electrical gradient across the phloem membrane (7), which drives the coupled H^+^/S transport via a co-transporter into the phloem (8), thereby dissipating the additional electrical gradient (9). In this model, the parenchyma cell remotely controls the transport across the neighboring phloem membrane with the actual objective of K^+^ uptake. For didactic reasons, our model shows the processes stepwise. In situ, however, the fluxes occur simultaneously. The membrane sandwich is a new, unique entity that has dynamic properties different from isolated membranes.

The transporters highlighted here are the main actors of the process. In fact, they are part of homeostats that adjust the steady-state conditions [23]. The homeostats are not shown in detail in order to focus on the essentials of the system. A general K-homeostat is built from K^+^ channels, K^+^/H^+^ symporters, and K^+^/H^+^ antiporters, while a general sugar homeostat is built from H^+^/sugar co-transporters and sugar diffusion facilitators (SWEETs). As stated initially, when starting the thought experiment, the different homeostats were in a steady state, meaning that all efflux via one transporter was compensated by an influx via another. Nutrients and ions thus cycle across the respective membranes [23]. The additional activity of the parenchyma H^+^-ATPase then breaks out of this balance. It should be noted that for the membrane sandwich coupling, it does not matter if the parenchyma cell accumulates K^+^ via a 1 H^+^/1 K^+^ exchange through K^+^ channels or via a 2 H^+^/1 K^+^/H^+^ exchange through proton-coupled K^+^ transporters. Nevertheless, the latter would cost the cell the hydrolysis of two ATP molecules, while the former would cost only one [24].

## 4. Example 2: Self-Regulatory Nutrient Trading in Mycorrhizal Symbioses

**Figure 3:**
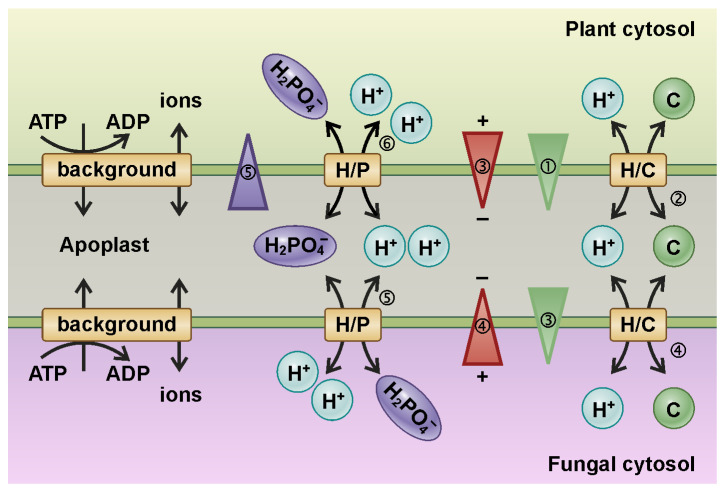
A minimal transporter system to explain the electrochemical coupling of nutrient transport at plant—fungi interfaces. For details, see the text. An animated version of this Figure can be found in the Supplementary Materials (Animation S2).

Remote control of transport processes at sandwich membranes was also deduced from the analysis of the interaction of plants with other organisms, in particular with fungi. In mycorrhizal associations, fungi colonize the root tissue of a host plant, either intracellularly, as in arbuscular mycorrhizal fungi (AMF or AM), or extracellularly, as in ectomycorrhizal fungi (ECM) [25,26,27,28,29]. In both cases, this creates a membrane sandwich structure of plant plasma membrane and fungal plasma membrane with a tiny apoplastic interorganismic space. The ECM hyphae stay away from the host cells and establish a sandwich of 500 nm–1 μm [30]. AM forms a much more intimate structure characterized by highly branched arbuscules, where fungal and plant membranes are separated by ~80–100 nm [8,31,32]. Plants and fungi exchange nutrients through this symbiotic interface, with the fungus providing phosphorus (P), nitrogen, and zinc, while the plant supplies reduced organic molecules (fixed carbon). The membrane interface exhibits particular dynamic features, as illustrated exemplarily by a minimal model describing the exchange of a phosphate (P) and a carbon (C) source [33] (Figure 3; Animation S2). One of the limiting factors for plant growth is the availability of P, while plants produce reduced carbon at low-cost thanks to photosynthesis. On the other hand, a limiting factor for fungal growth is the lower availability of carbon, while fungi can absorb P very efficiently. At the interface between plants and fungi, an exchange takes place, with the plant providing a C source and exchanging C for P provided by the fungus. The C–P trade is governed by the coupling of transport across both membranes.

To illustrate this coupling mechanism, we assume that the system is initially in a steady state, i.e., there is no net transport across either membrane. In the following, the different processes are again presented step-by-step. (1) As a result of its photosynthetic activity, the plant is able to increase the cytosolic C concentration and thereby establish an additional contribution to the [C] gradient across the plant membrane. (2) This gradient energizes the coupled H^+^/C-efflux via a C homeostat (H/C) [23], which renders the electric potential in the plant cytosol more negative due to the charge transport (3). The increase [C] in the apoplast causes an additional contribution to the [C] gradient across the fungal membrane, which in turn drives the coupled influx of H^+^ and C into the fungus (4). This transmembrane charge transport establishes an additional contribution to the electric gradient across the fungal membrane (4), which energizes the coupled 2 H^+^/1 H_2_PO_4_^−^ efflux from the fungus to the apoplast via an H^+^-coupled H_2_PO_4_^−^ transporter (5). The increased [P] in the apoplast creates an additional contribution to the [P] gradient across the plant membrane. In addition to the so far not compensated additional electric gradient (3), the P gradient further energizes the coupled 2 H^+^/1 H_2_PO_4_^−^ uptake by the plant (6). As in the model of the phloem sandwich described above, these different steps occur simultaneously and are not separate for each membrane, pointing to new, unique dynamic properties of the membrane sandwich.

The presented example of an increase in [C] in the plant can be repeated analogously with changes of [C] and [P] in the plant and the fungus, with corresponding results. An increase in [P] in the fungus stimulates a larger P-flux from the fungus to the plant and a larger C-flux in the reverse direction [33]. The link between the C- and P-fluxes due to the dynamics of the membrane sandwich explains, straightforwardly, the observation of reciprocal rewards that stabilize the symbiosis between the plant and fungus [34]. The minimal model can also be expanded to approach more complex physiological contexts [28,35]. For instance, the transport of nitrogen sources can also be included [35]. However, regardless of how complex the model is designed, the electrochemical coupling of the two membranes via the concentration in the apoplast and the transmembrane charge transport remains the fundamental principle that rules the novel features of the membrane sandwich module.

In addition to explaining the basics of the mutual nutrient deal between the plant and fungus as a self-organizing process, mathematical/thermodynamic modeling of the transport processes at sandwich membranes enabled further fundamental physiological insights. With the available experimental techniques, it is extremely difficult to determine the concentrations in the apoplast of the symbiotic interface. Model calculations suggest that one consequence of the coupling of the two membranes is nutrient concentrations that are much lower than generally assumed [33,36]. Indeed, low concentrations in the apoplast are a prerequisite for the rapid and sensitive perception of changes caused by the interaction partner, which minimizes the risk of being cheated in the symbiotic interaction.

## 5. Example 3: A Small Step from Mutualism to Parasitism in Plant–Fungus Interactions

**Figure 4:**
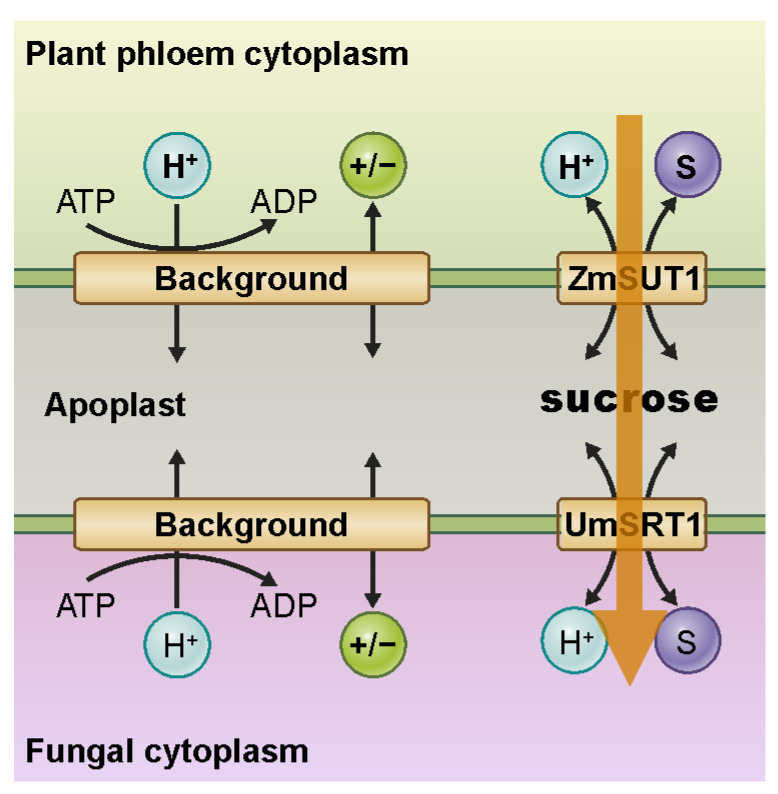
Nutrient transport at plant fungi interfaces in a parasitic relationship. For details, see the text. An animation illustrating more details of this model system can be found in the Supplementary Materials (Animation S3).

Fungi can not only interact symbiotically with plants. In many cases, they show parasitic behavior instead. A typical example is the fungus *Ustilago maydis*, which causes corn smut disease and induces tumor formation in its host *Zea mays* [37]. During infection, the fungal hyphae meshwork grows selectively along the phloem vessels [38] and creates a contact zone near the sugar-conducting cells of the sieve element companion cell (SE/CC) complex. *Ustilago maydis* hyphae can establish strong interaction with their host, entering into cells and forming sandwich structures with intramembrane compartments in the range of 100 nm [39] (Figure 4; Animation S3). Interestingly, models similar to those used to describe mycorrhizal symbiosis [33,35] (Figure 3) could also be applied here, illustrating the fine line between mutualism and parasitism [40].

The symbiotic nutrient exchange between plant and fungus works as long as both participants are energetically on equal terms [33]. In the root cortex, this is often the case. In phloem tissue, however, the interaction shows a parasitic nature. Maintaining the [C] gradient in the phloem is necessary to ensure bulk flow from source to sink, but it represents an Achilles’ heel that fungi exploit to absorb [C] through a membrane sandwich. Both the H^+^-ATPase, as well as a potassium gradient, are needed to energize the membrane and to allow the establishment of a high [C] gradient [14,15,16]. However, critical energy conditions make any structure vulnerable to attack from the outside, which is apparent in this case as well. The high [C] concentration in the cytosol of the plant does not allow the [C] concentration in the apoplast to be reduced to very low levels, as is the case, for example, in mycorrhizal symbiosis. The plant simply does not have the energy resources necessary in this case to compete with the fungus for the nutrient source. The missing competition uncouples the different transport processes at the membrane sandwich. At the fungal side of the sandwich, the [C] gradient across the fungal membrane is much lower than on the phloem side because the cytosolic [C] of the fungus is not as high as in the phloem. As a consequence, the fungus benefits from the C source in the apoplast, the absorption of which is not associated with the delivery of P or other nutrients, as in a symbiotic relationship (Figure 4). Rather, the fungus must ensure, through special adaptation of its transporter properties, that C uptake does not occur too rapidly and potentially cause undesirable osmotic side effects [40] (Animation S3). An unusually high affinity of the fungal H^+^/sugar co-transporter guarantees that this transporter operates at its saturation limit. Therefore, a sudden increase in apoplastic sugar concentration, as can occur due to a leak in the plant phloem, does not lead to an increased transport capacity across the fungal membrane and flooding of the fungal cytosol with osmotically problematic sucrose.

This example illustrates that organisms have adapted to the special dynamics of membrane sandwiches. Interestingly, in wheat, it has been demonstrated how a plant could counterstrike in this type of arms race. Partial resistance to fungal pathogens was achieved by mutations in a sugar transporter that render it incapable of transporting hexoses [41]. This handicap apparently reduces sugar leakage from the phloem and reduces its availability to fungi.

## 6. Conclusions and Outlook

Membrane sandwiches are omnipresent in plants. Yet, they seem to have received little attention. Here, we consider these morphological structures as important for physiological functions and demonstrate unique properties emerging from membrane sandwiches. Meanwhile, there are first reports on the communication between the plasma membrane and the vacuolar membrane [42,43]. However, the far-reaching significance of dual-membrane systems still does not seem to have been fully recognized. On the contrary, sometimes their importance seems to be artificially belittled [43]. The conclusion that, for example, the semi-open nature of the apoplast, i.e., that it is not a tightly closed compartment, may moderate the interactions between membranes largely neglects the actual geometry of the systems. A contact zone between two plasma membranes can extend over hundreds of μm^2^. This means that the 1 μm^2^ patch we originally considered in the second paragraph is surrounded by other identical patches in all directions. At the immediate edges of the contact zone, dilution effects may well occur. However, these dilution effects are not present in the core of the contact zone. There, the transport processes across one membrane affect the transport across the other without significant dissipation. Thus, the contact zones are special and may be characterized by specifically adapted expression of membrane transporters.

In the intermembrane compartment of the sandwich of two plasma membranes, charged polymers from the cell wall may interact with ions, especially protons and divalent cations, such as Ca^2+^. A pectin methylesterase enzyme adds charges to pectin homogalacturonans upon activation and alters the local Ca^2+^ and H^+^ buffer capacity of the compartment [44,45]. With respect to pH, lateral homogeneity and transverse gradients have been observed, supporting the idea that biochemical activity at the cell wall may affect the electrochemical properties of the compartment, free ion concentration, and electrodiffusion [46,47]. These findings suggest that the pH of the cell wall reflects not only the activity of the proton pump and ion channels at the plasma membrane, but also the buffering capacity of polyanionic cell wall polymers. Consequently, the cell wall has to be considered as an additional player in the dynamics of membrane sandwiches involving two plasma membranes.

In this article, we present a few glimpses of the surprising dynamics of membrane sandwiches in plants. At this stage, we are far from grasping the full extent of such phenomena. In the future, we all will need to strengthen our efforts in the investigation of the systemic character of membrane transport processes. Considering those limits, we offer a new conceptual framework that we applied on the basis of the thermodynamics of transport. Membrane sandwiches constitute physiological interfaces that control most plant growth through photo assimilate allocations and plant interaction with pathogens and symbionts. By consistently following this new path, we may be able to develop unprecedented strategies to improve salt and drought tolerance, and nutrient use efficiency of crops.

## Data Availability

All data are included in the manuscript. Additional information is available upon request from the corresponding authors.

## Supplementary Materials

The following supporting information can be downloaded at https://www.mdpi.com/article/10.3390/plants12010204/s1, Animation S1: Remote Control of Sugar Uptake; Animation S2: Nutrient Trade in Mycorrhizal Symbiosis; and Animation S3: From symbiosis to parasitism. This material package was designed specifically to support teaching at the undergraduate and graduate levels.
