# Supplementary material for: The Surprising Dynamics of Electrochemical Coupling at Membrane Sandwiches in Plants
Source: Plants (Basel). 2023 Jan 3;12(1):204. doi: 10.3390/plants12010204 (PMC9824766; doi:10.3390/plants12010204)
Supplement: Supplementary file 1 [file plants-12-00204-s001.zip › plants-2098129-supplementary/Animation_S1.pptx]

## Slide 1
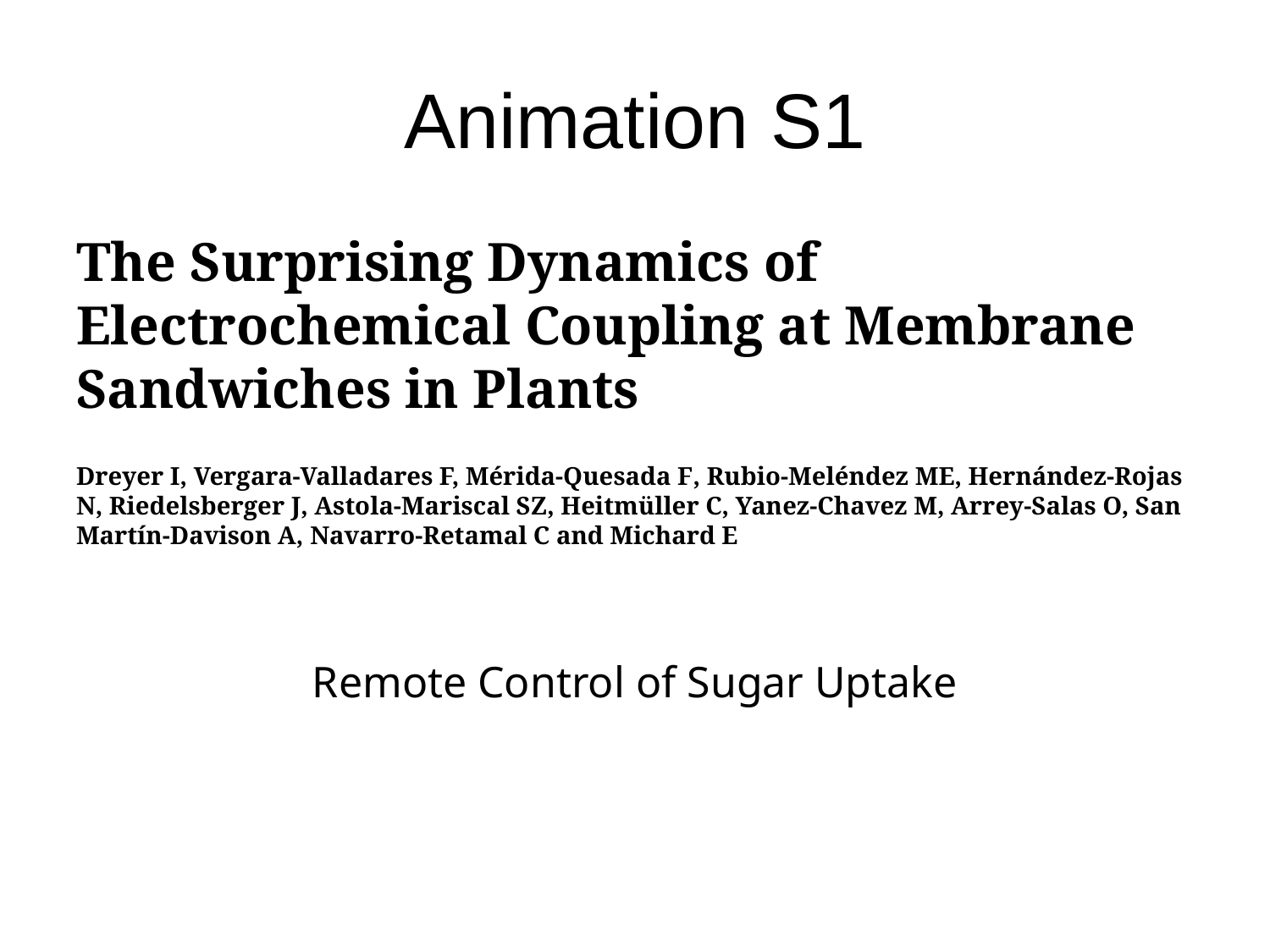

# Animation S1
The Surprising Dynamics of Electrochemical Coupling at Membrane Sandwiches in Plants
Dreyer I, Vergara-Valladares F, Mérida-Quesada F, Rubio-Meléndez ME, Hernández-Rojas N, Riedelsberger J, Astola-Mariscal SZ, Heitmüller C, Yanez-Chavez M, Arrey-Salas O, San Martín-Davison A, Navarro-Retamal C and Michard E
Remote Control of Sugar Uptake

## Slide 2
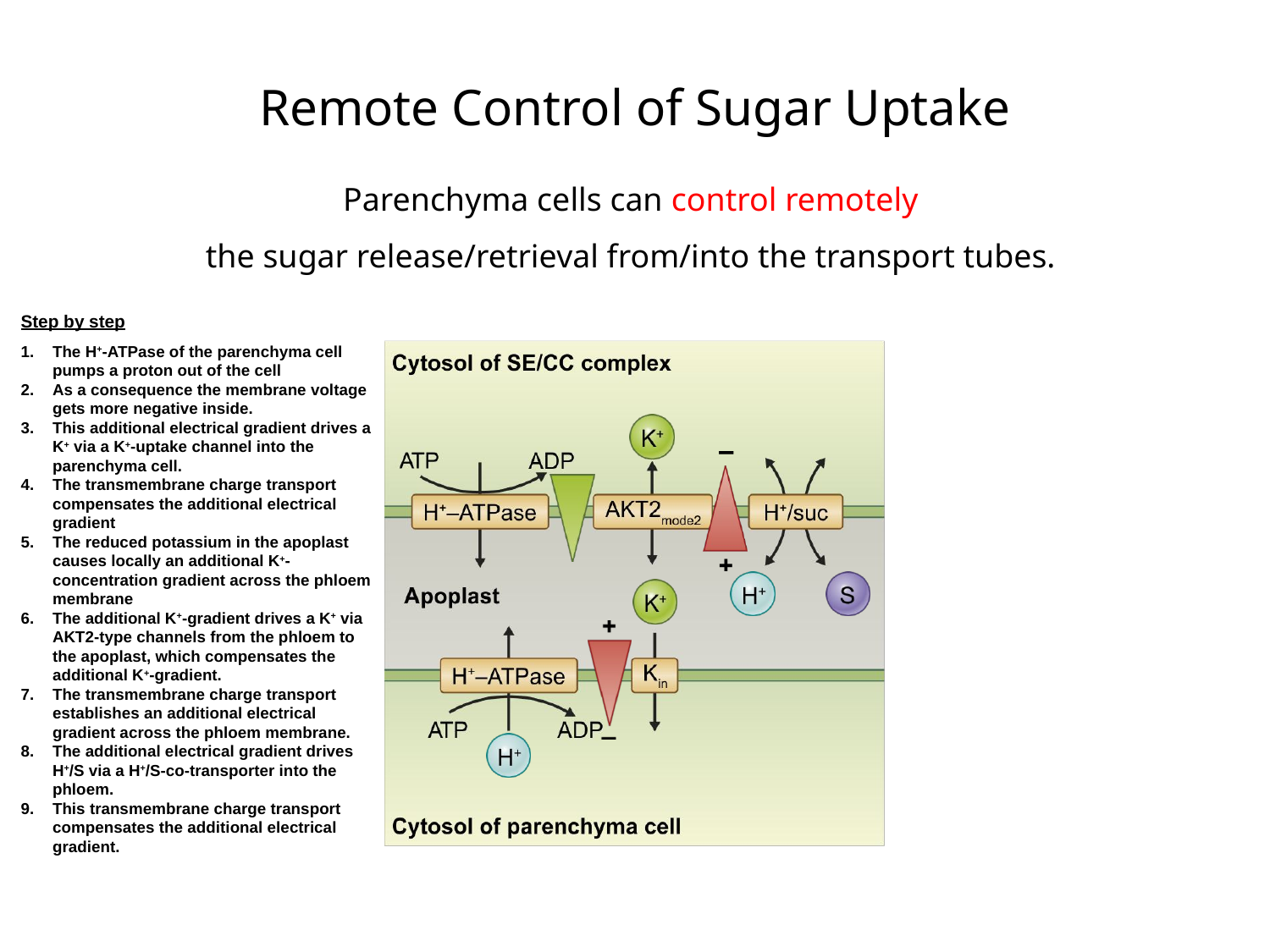

Remote Control of Sugar Uptake
Parenchyma cells can control remotely
the sugar release/retrieval from/into the transport tubes.
Step by step
The H+-ATPase of the parenchyma cell pumps a proton out of the cell
As a consequence the membrane voltage gets more negative inside.
This additional electrical gradient drives a K+ via a K+-uptake channel into the parenchyma cell.
The transmembrane charge transport compensates the additional electrical gradient
The reduced potassium in the apoplast causes locally an additional K+-concentration gradient across the phloem membrane
The additional K+-gradient drives a K+ via AKT2-type channels from the phloem to the apoplast, which compensates the additional K+-gradient.
The transmembrane charge transport establishes an additional electrical gradient across the phloem membrane.
The additional electrical gradient drives H+/S via a H+/S-co-transporter into the phloem.
This transmembrane charge transport compensates the additional electrical gradient.

## Slide 3
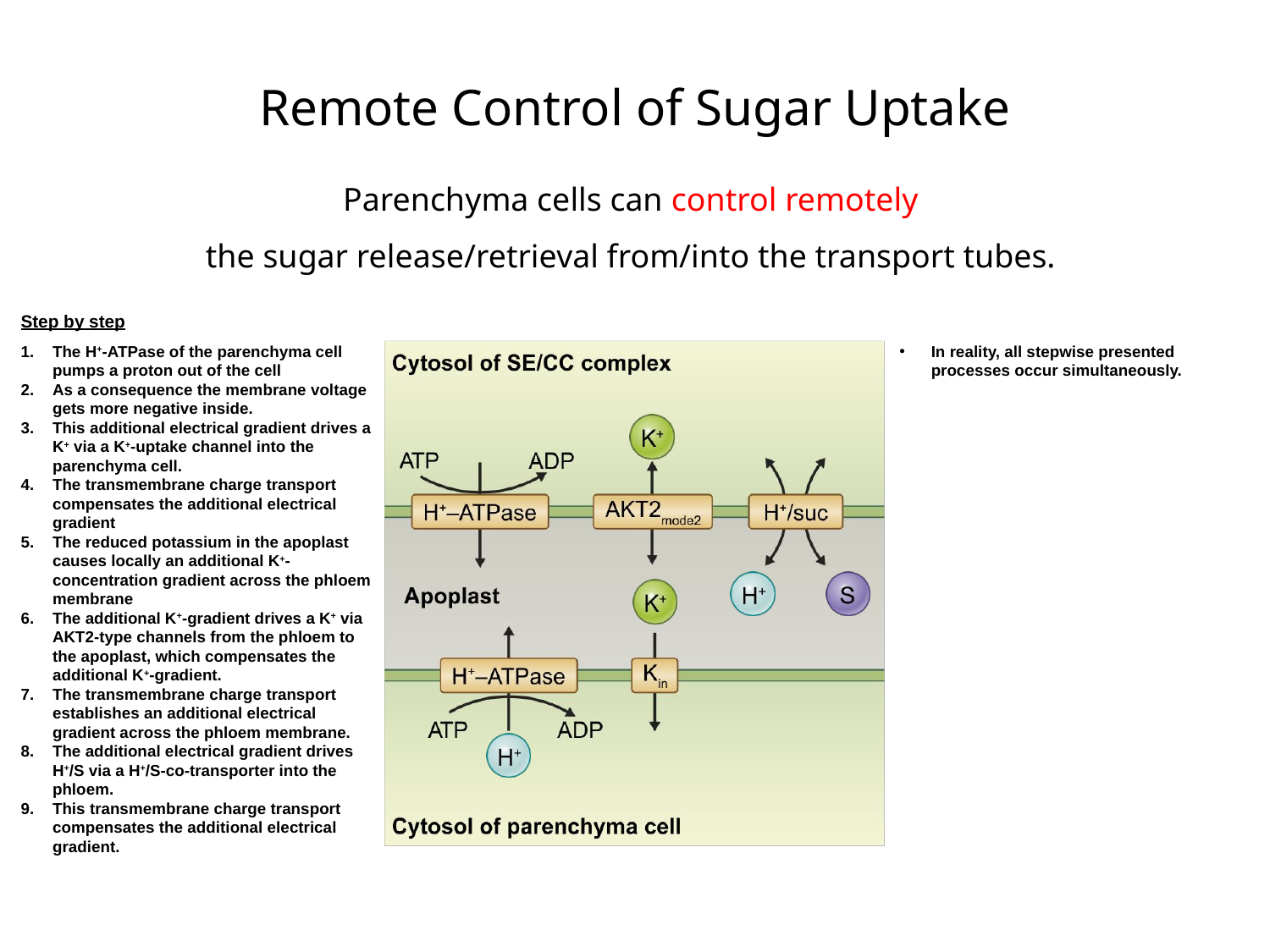

Remote Control of Sugar Uptake
Parenchyma cells can control remotely
the sugar release/retrieval from/into the transport tubes.
Step by step
The H+-ATPase of the parenchyma cell pumps a proton out of the cell
As a consequence the membrane voltage gets more negative inside.
This additional electrical gradient drives a K+ via a K+-uptake channel into the parenchyma cell.
The transmembrane charge transport compensates the additional electrical gradient
The reduced potassium in the apoplast causes locally an additional K+-concentration gradient across the phloem membrane
The additional K+-gradient drives a K+ via AKT2-type channels from the phloem to the apoplast, which compensates the additional K+-gradient.
The transmembrane charge transport establishes an additional electrical gradient across the phloem membrane.
The additional electrical gradient drives H+/S via a H+/S-co-transporter into the phloem.
This transmembrane charge transport compensates the additional electrical gradient.
In reality, all stepwise presented processes occur simultaneously.

## Slide 4
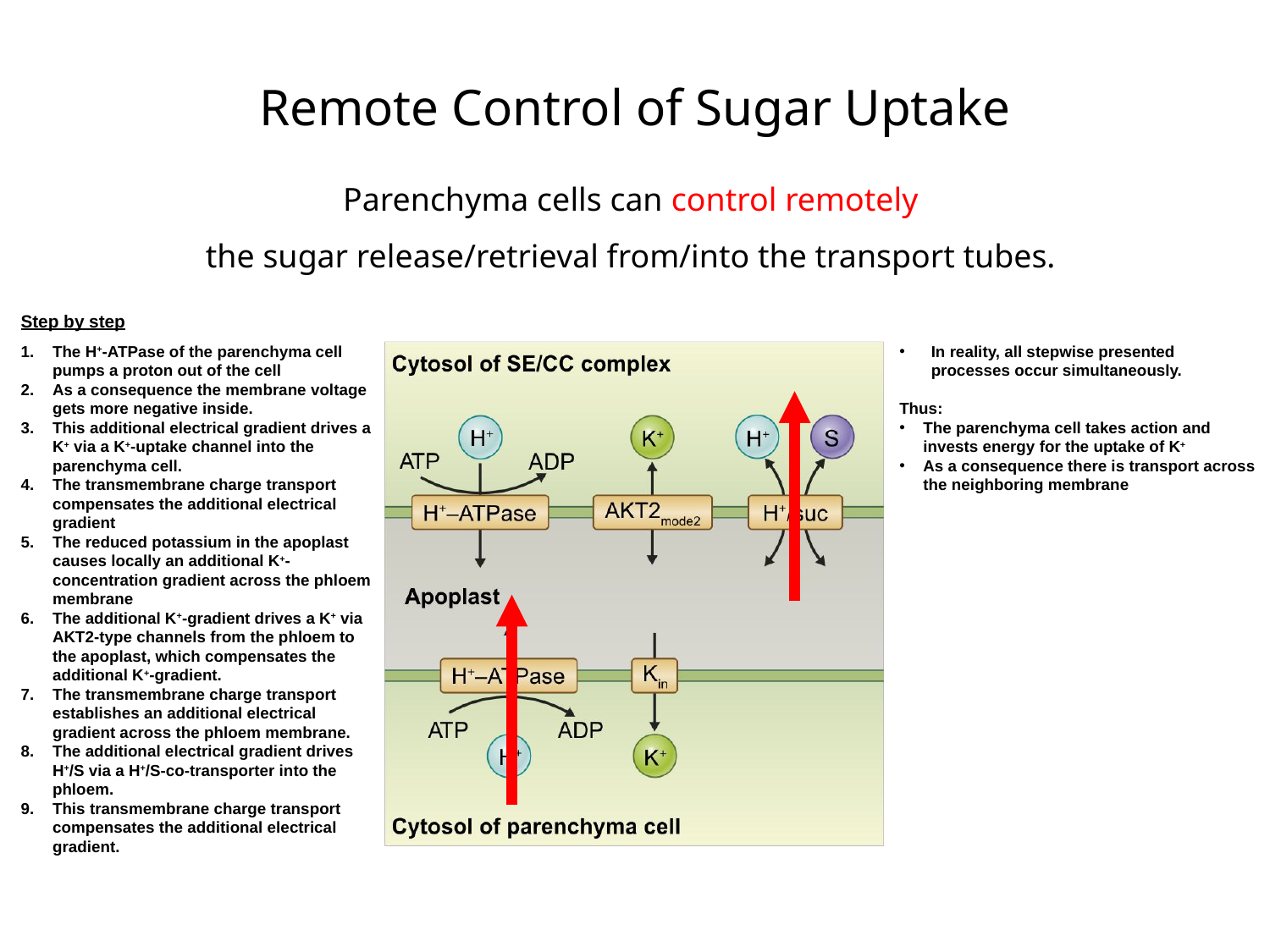

Remote Control of Sugar Uptake
Parenchyma cells can control remotely
the sugar release/retrieval from/into the transport tubes.
Step by step
The H+-ATPase of the parenchyma cell pumps a proton out of the cell
As a consequence the membrane voltage gets more negative inside.
This additional electrical gradient drives a K+ via a K+-uptake channel into the parenchyma cell.
The transmembrane charge transport compensates the additional electrical gradient
The reduced potassium in the apoplast causes locally an additional K+-concentration gradient across the phloem membrane
The additional K+-gradient drives a K+ via AKT2-type channels from the phloem to the apoplast, which compensates the additional K+-gradient.
The transmembrane charge transport establishes an additional electrical gradient across the phloem membrane.
The additional electrical gradient drives H+/S via a H+/S-co-transporter into the phloem.
This transmembrane charge transport compensates the additional electrical gradient.
In reality, all stepwise presented processes occur simultaneously.
Thus:
The parenchyma cell takes action and invests energy for the uptake of K+
As a consequence there is transport across the neighboring membrane

## Slide 5
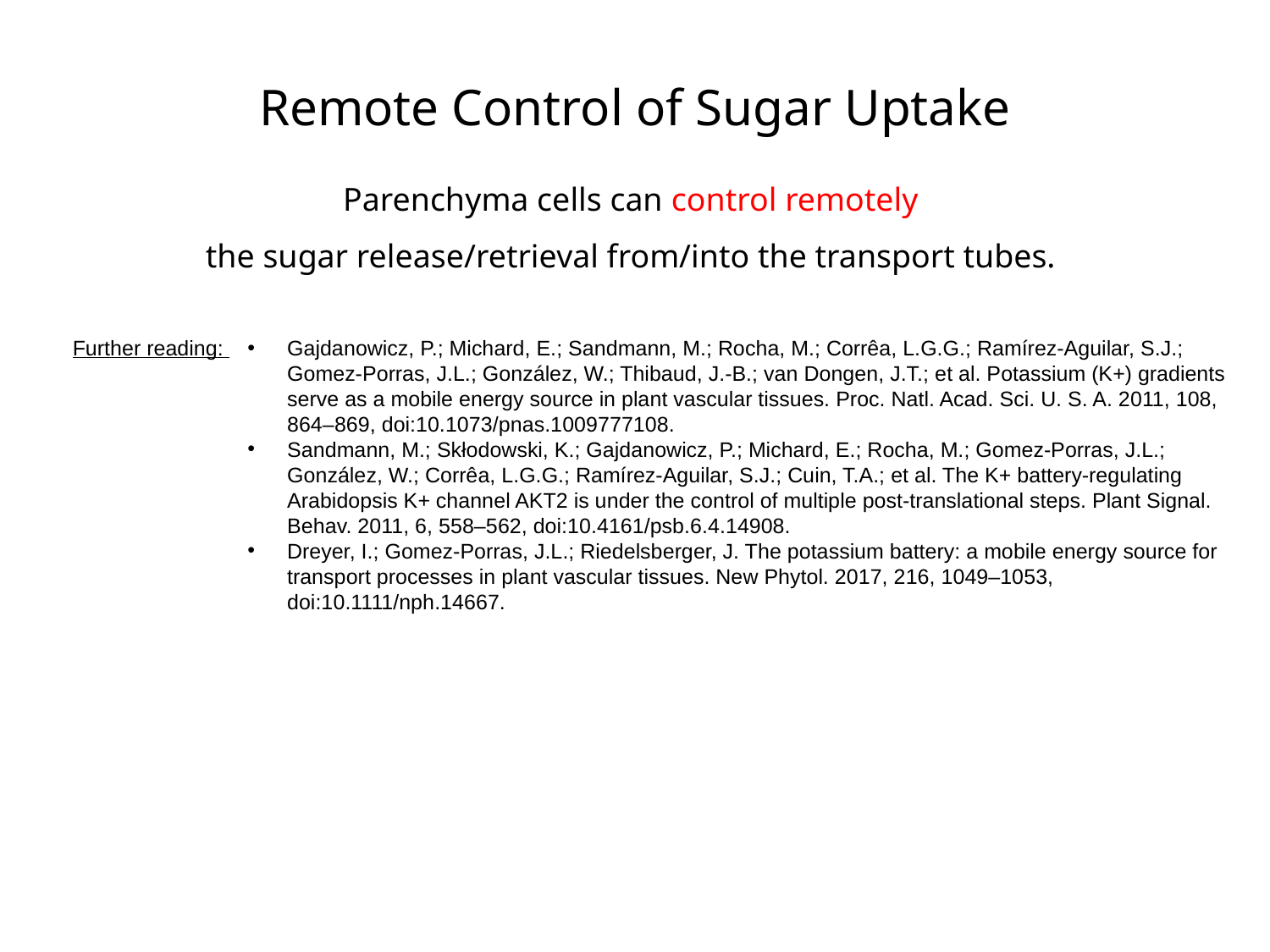

Remote Control of Sugar Uptake
Parenchyma cells can control remotely
the sugar release/retrieval from/into the transport tubes.
Further reading:
Gajdanowicz, P.; Michard, E.; Sandmann, M.; Rocha, M.; Corrêa, L.G.G.; Ramírez-Aguilar, S.J.; Gomez-Porras, J.L.; González, W.; Thibaud, J.-B.; van Dongen, J.T.; et al. Potassium (K+) gradients serve as a mobile energy source in plant vascular tissues. Proc. Natl. Acad. Sci. U. S. A. 2011, 108, 864–869, doi:10.1073/pnas.1009777108.
Sandmann, M.; Skłodowski, K.; Gajdanowicz, P.; Michard, E.; Rocha, M.; Gomez-Porras, J.L.; González, W.; Corrêa, L.G.G.; Ramírez-Aguilar, S.J.; Cuin, T.A.; et al. The K+ battery-regulating Arabidopsis K+ channel AKT2 is under the control of multiple post-translational steps. Plant Signal. Behav. 2011, 6, 558–562, doi:10.4161/psb.6.4.14908.
Dreyer, I.; Gomez-Porras, J.L.; Riedelsberger, J. The potassium battery: a mobile energy source for transport processes in plant vascular tissues. New Phytol. 2017, 216, 1049–1053, doi:10.1111/nph.14667.
