# Supplementary material for: The Surprising Dynamics of Electrochemical Coupling at Membrane Sandwiches in Plants
Source: Plants (Basel). 2023 Jan 3;12(1):204. doi: 10.3390/plants12010204 (PMC9824766; doi:10.3390/plants12010204)
Supplement: Supplementary file 1 [file plants-12-00204-s001.zip › plants-2098129-supplementary/Animation_S2.pptx]

## Slide 1
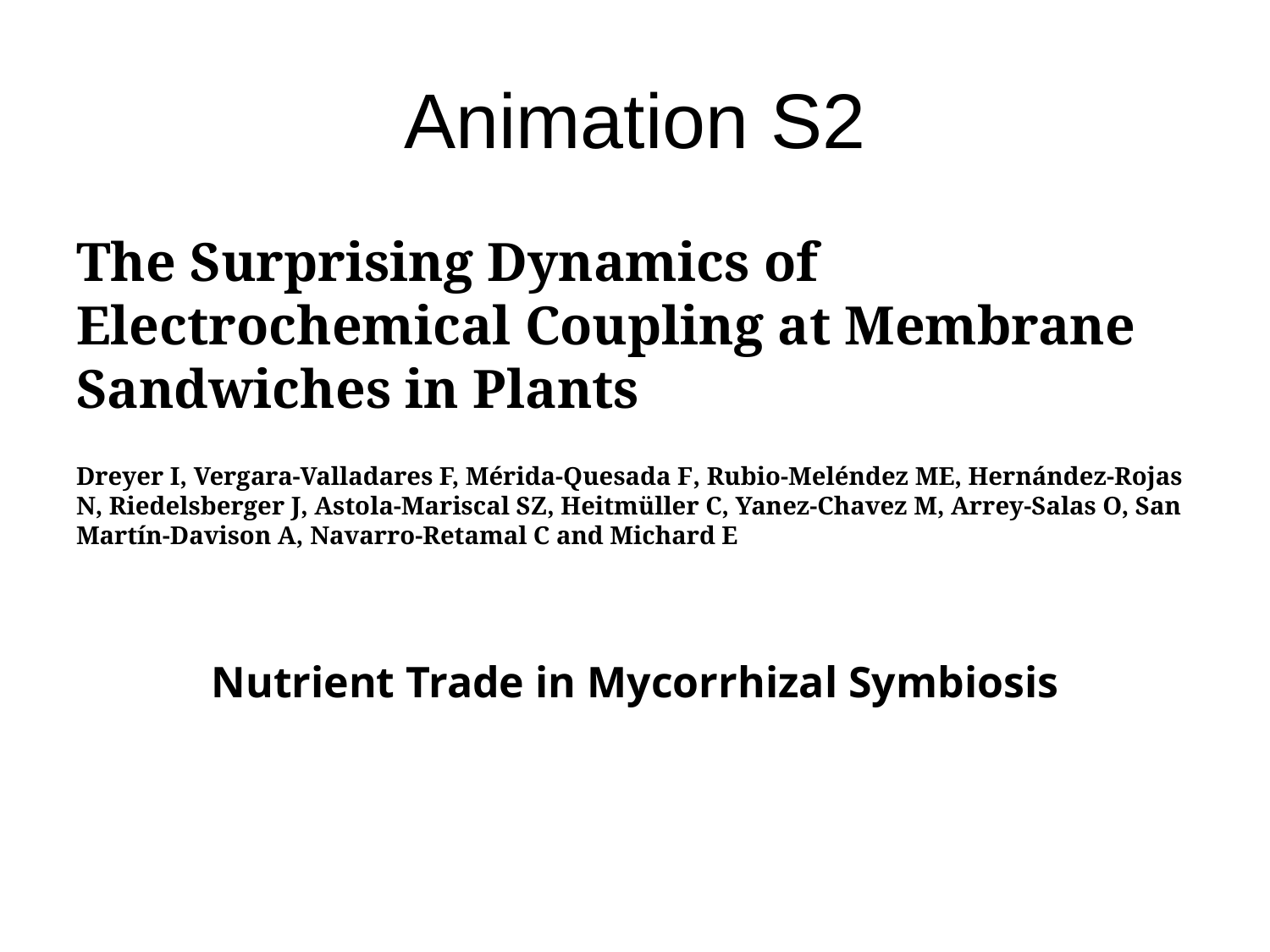

# Animation S2
The Surprising Dynamics of Electrochemical Coupling at Membrane Sandwiches in Plants
Dreyer I, Vergara-Valladares F, Mérida-Quesada F, Rubio-Meléndez ME, Hernández-Rojas N, Riedelsberger J, Astola-Mariscal SZ, Heitmüller C, Yanez-Chavez M, Arrey-Salas O, San Martín-Davison A, Navarro-Retamal C and Michard E
Nutrient Trade in Mycorrhizal Symbiosis

## Slide 2
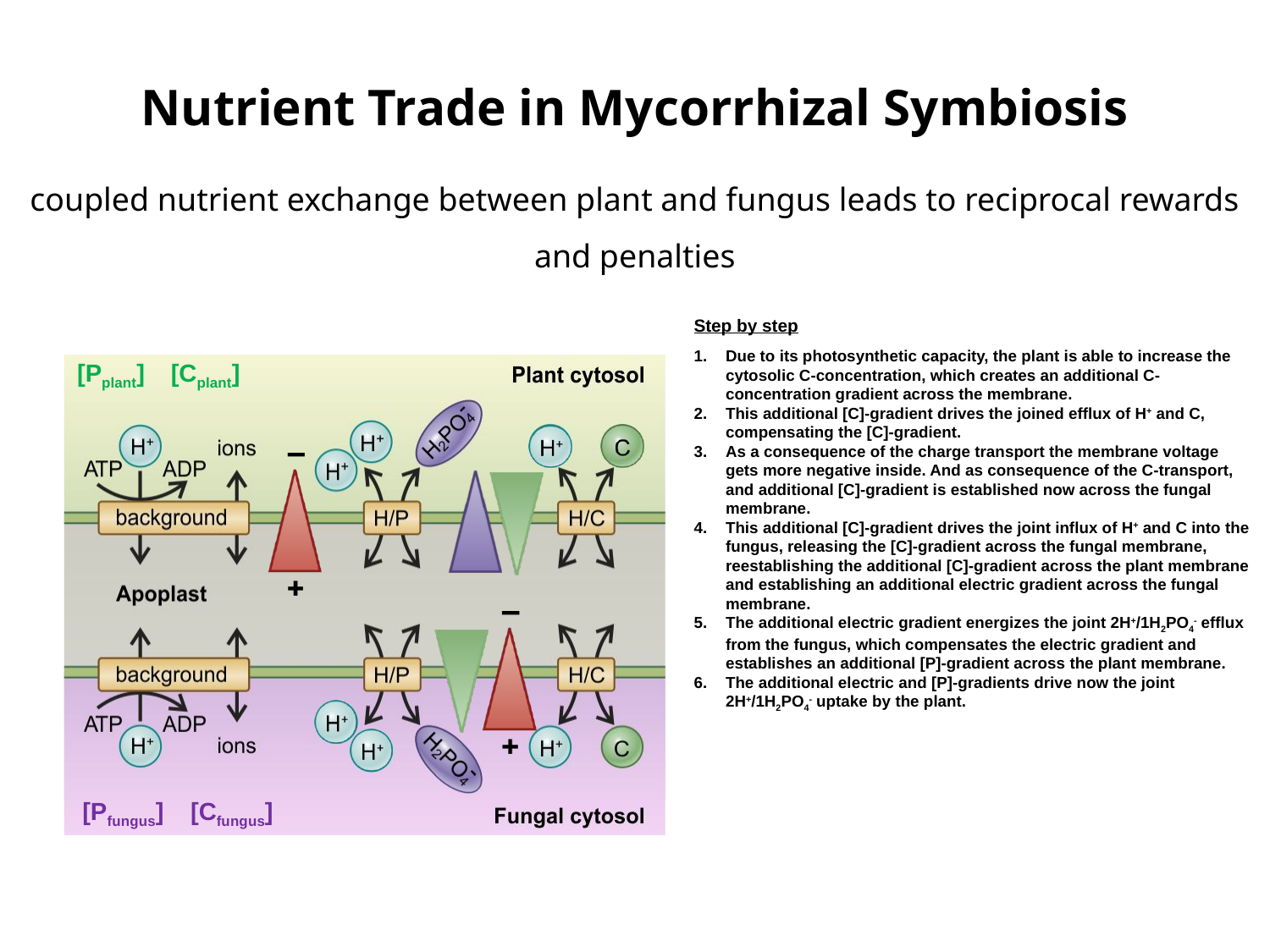

Nutrient Trade in Mycorrhizal Symbiosis
coupled nutrient exchange between plant and fungus leads to reciprocal rewards and penalties
Step by step
Due to its photosynthetic capacity, the plant is able to increase the cytosolic C-concentration, which creates an additional C-concentration gradient across the membrane.
This additional [C]-gradient drives the joined efflux of H+ and C, compensating the [C]-gradient.
As a consequence of the charge transport the membrane voltage gets more negative inside. And as consequence of the C-transport, and additional [C]-gradient is established now across the fungal membrane.
This additional [C]-gradient drives the joint influx of H+ and C into the fungus, releasing the [C]-gradient across the fungal membrane, reestablishing the additional [C]-gradient across the plant membrane and establishing an additional electric gradient across the fungal membrane.
The additional electric gradient energizes the joint 2H+/1H2PO4- efflux from the fungus, which compensates the electric gradient and establishes an additional [P]-gradient across the plant membrane.
The additional electric and [P]-gradients drive now the joint 2H+/1H2PO4- uptake by the plant.
[Pplant]
[Cplant]
[Pfungus]
[Cfungus]

## Slide 3
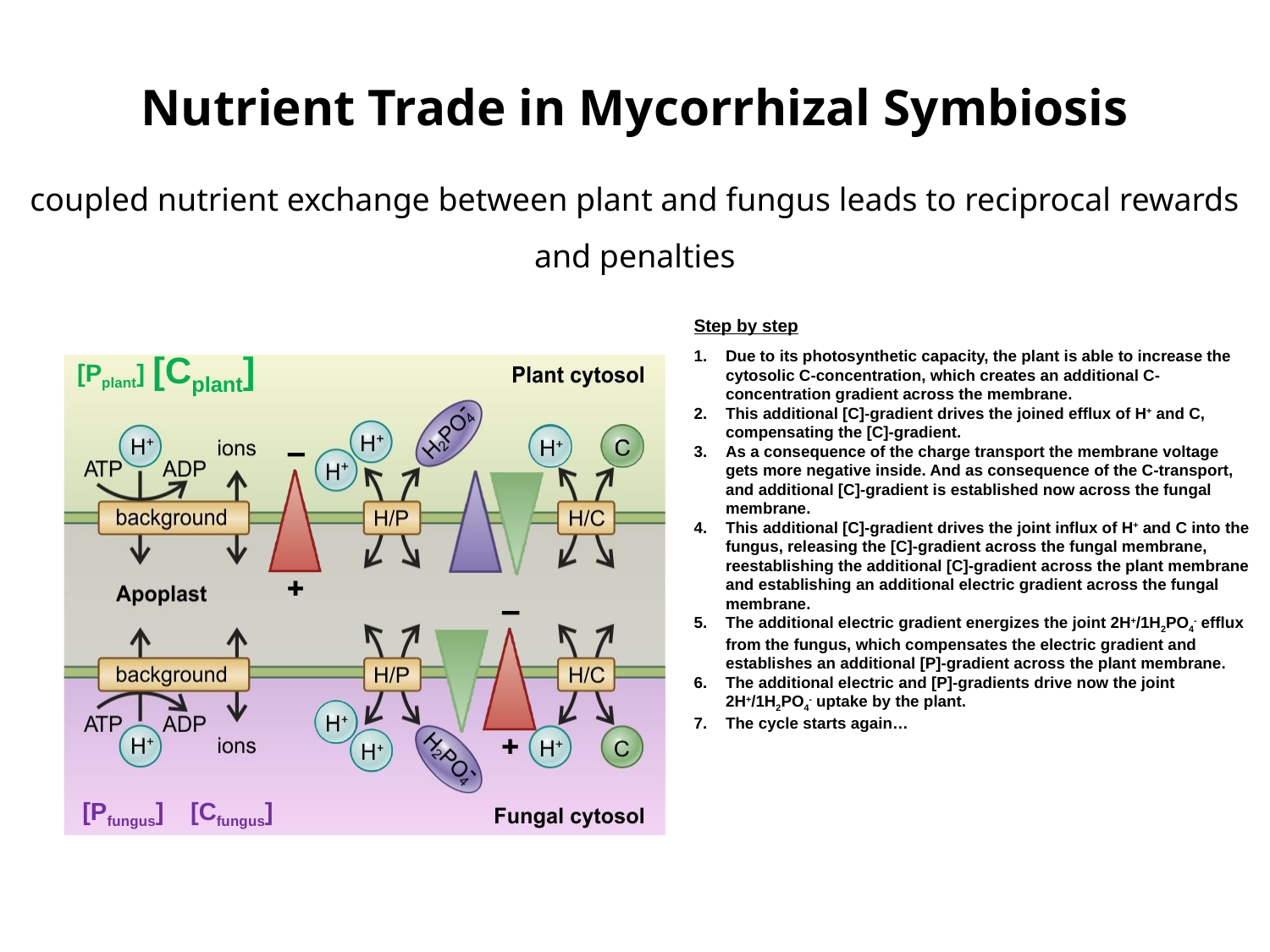

Nutrient Trade in Mycorrhizal Symbiosis
coupled nutrient exchange between plant and fungus leads to reciprocal rewards and penalties
Step by step
Due to its photosynthetic capacity, the plant is able to increase the cytosolic C-concentration, which creates an additional C-concentration gradient across the membrane.
This additional [C]-gradient drives the joined efflux of H+ and C, compensating the [C]-gradient.
As a consequence of the charge transport the membrane voltage gets more negative inside. And as consequence of the C-transport, and additional [C]-gradient is established now across the fungal membrane.
This additional [C]-gradient drives the joint influx of H+ and C into the fungus, releasing the [C]-gradient across the fungal membrane, reestablishing the additional [C]-gradient across the plant membrane and establishing an additional electric gradient across the fungal membrane.
The additional electric gradient energizes the joint 2H+/1H2PO4- efflux from the fungus, which compensates the electric gradient and establishes an additional [P]-gradient across the plant membrane.
The additional electric and [P]-gradients drive now the joint 2H+/1H2PO4- uptake by the plant.
The cycle starts again…
[Cplant]
[Pplant]
[Pfungus]
[Cfungus]

## Slide 4
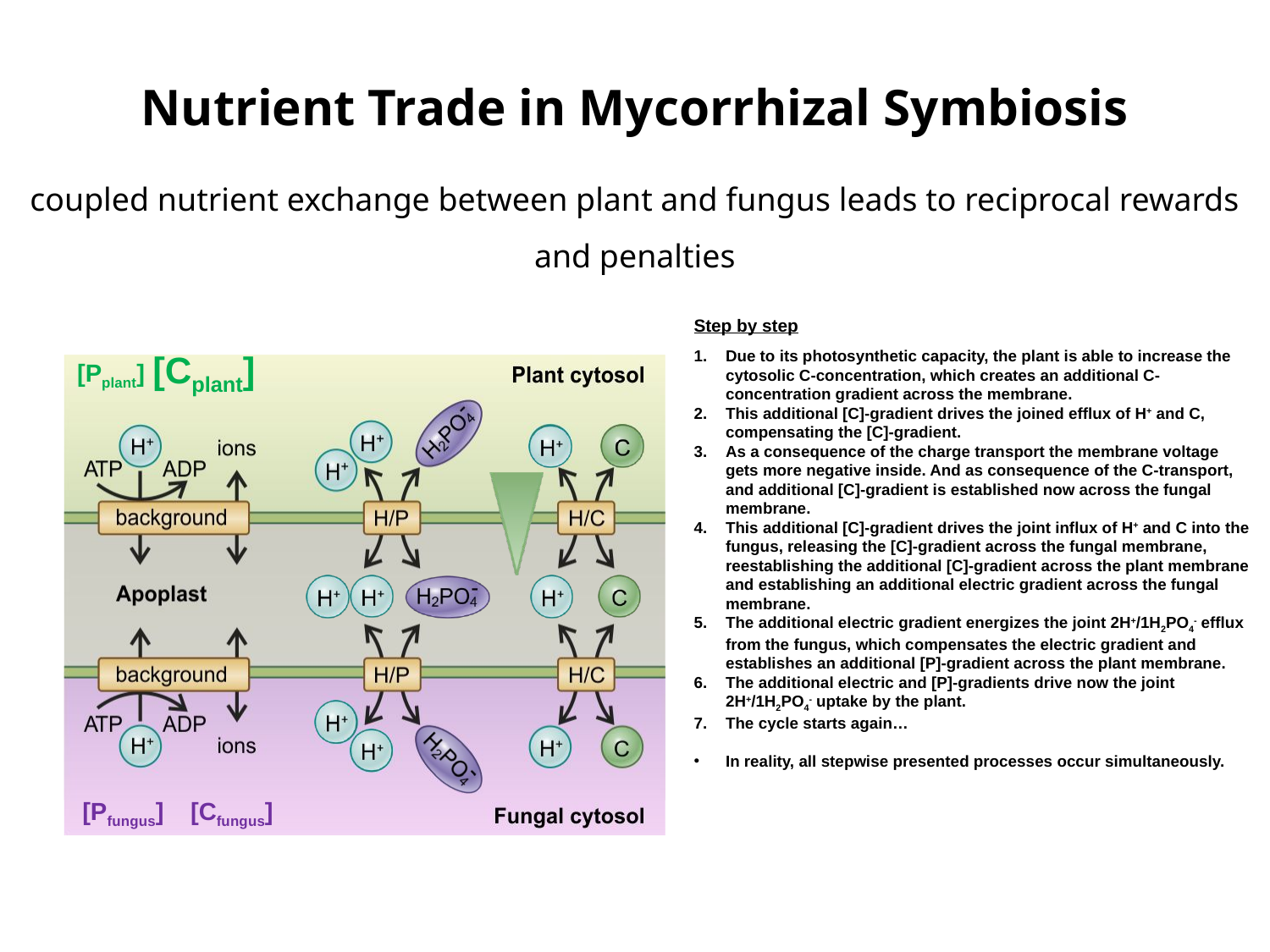

Nutrient Trade in Mycorrhizal Symbiosis
coupled nutrient exchange between plant and fungus leads to reciprocal rewards and penalties
Step by step
Due to its photosynthetic capacity, the plant is able to increase the cytosolic C-concentration, which creates an additional C-concentration gradient across the membrane.
This additional [C]-gradient drives the joined efflux of H+ and C, compensating the [C]-gradient.
As a consequence of the charge transport the membrane voltage gets more negative inside. And as consequence of the C-transport, and additional [C]-gradient is established now across the fungal membrane.
This additional [C]-gradient drives the joint influx of H+ and C into the fungus, releasing the [C]-gradient across the fungal membrane, reestablishing the additional [C]-gradient across the plant membrane and establishing an additional electric gradient across the fungal membrane.
The additional electric gradient energizes the joint 2H+/1H2PO4- efflux from the fungus, which compensates the electric gradient and establishes an additional [P]-gradient across the plant membrane.
The additional electric and [P]-gradients drive now the joint 2H+/1H2PO4- uptake by the plant.
The cycle starts again…
In reality, all stepwise presented processes occur simultaneously.
[Cplant]
[Pplant]
[Pfungus]
[Cfungus]

## Slide 5
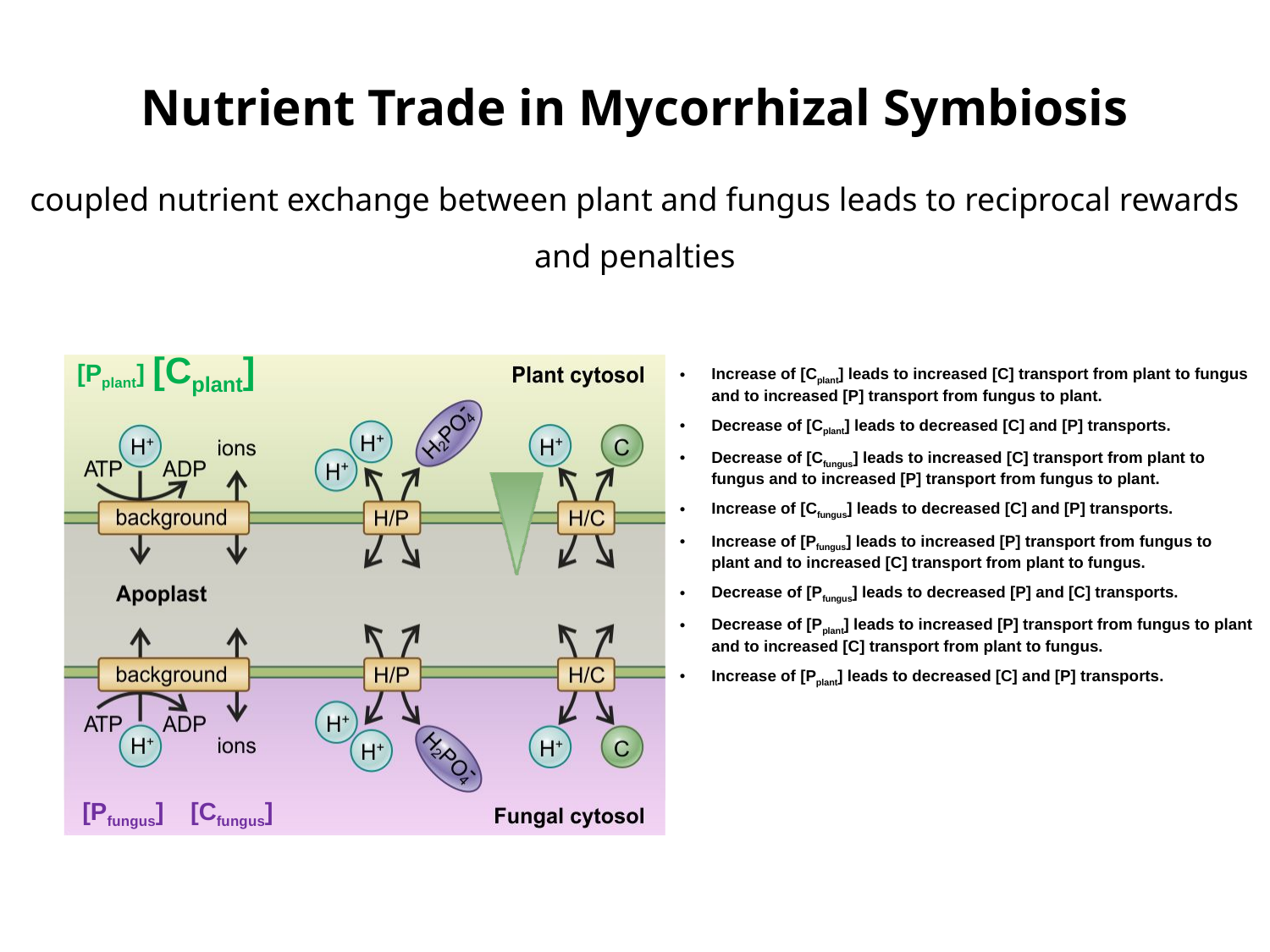

Nutrient Trade in Mycorrhizal Symbiosis
coupled nutrient exchange between plant and fungus leads to reciprocal rewards and penalties
[Cplant]
[Pplant]
Increase of [Cplant] leads to increased [C] transport from plant to fungus and to increased [P] transport from fungus to plant.
Decrease of [Cplant] leads to decreased [C] and [P] transports.
Decrease of [Cfungus] leads to increased [C] transport from plant to fungus and to increased [P] transport from fungus to plant.
Increase of [Cfungus] leads to decreased [C] and [P] transports.
Increase of [Pfungus] leads to increased [P] transport from fungus to plant and to increased [C] transport from plant to fungus.
Decrease of [Pfungus] leads to decreased [P] and [C] transports.
Decrease of [Pplant] leads to increased [P] transport from fungus to plant and to increased [C] transport from plant to fungus.
Increase of [Pplant] leads to decreased [C] and [P] transports.
[Pfungus]
[Cfungus]

## Slide 6
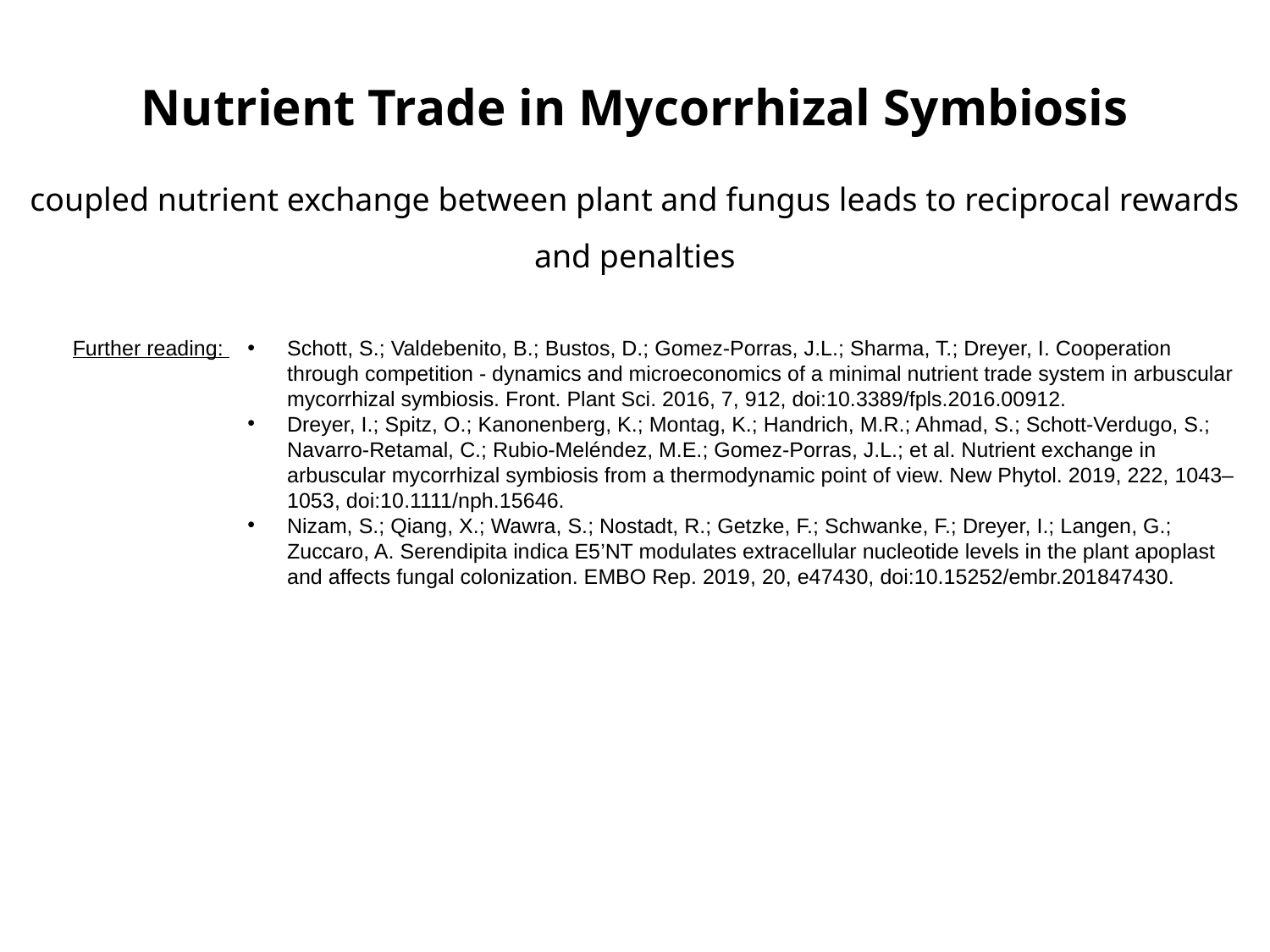

Nutrient Trade in Mycorrhizal Symbiosis
coupled nutrient exchange between plant and fungus leads to reciprocal rewards and penalties
Further reading:
Schott, S.; Valdebenito, B.; Bustos, D.; Gomez-Porras, J.L.; Sharma, T.; Dreyer, I. Cooperation through competition - dynamics and microeconomics of a minimal nutrient trade system in arbuscular mycorrhizal symbiosis. Front. Plant Sci. 2016, 7, 912, doi:10.3389/fpls.2016.00912.
Dreyer, I.; Spitz, O.; Kanonenberg, K.; Montag, K.; Handrich, M.R.; Ahmad, S.; Schott‐Verdugo, S.; Navarro‐Retamal, C.; Rubio‐Meléndez, M.E.; Gomez‐Porras, J.L.; et al. Nutrient exchange in arbuscular mycorrhizal symbiosis from a thermodynamic point of view. New Phytol. 2019, 222, 1043–1053, doi:10.1111/nph.15646.
Nizam, S.; Qiang, X.; Wawra, S.; Nostadt, R.; Getzke, F.; Schwanke, F.; Dreyer, I.; Langen, G.; Zuccaro, A. Serendipita indica E5’NT modulates extracellular nucleotide levels in the plant apoplast and affects fungal colonization. EMBO Rep. 2019, 20, e47430, doi:10.15252/embr.201847430.
