# Supplementary material for: The Surprising Dynamics of Electrochemical Coupling at Membrane Sandwiches in Plants
Source: Plants (Basel). 2023 Jan 3;12(1):204. doi: 10.3390/plants12010204 (PMC9824766; doi:10.3390/plants12010204)
Supplement: Supplementary file 1 [file plants-12-00204-s001.zip › plants-2098129-supplementary/Animation_S3.pptx]

## Slide 1
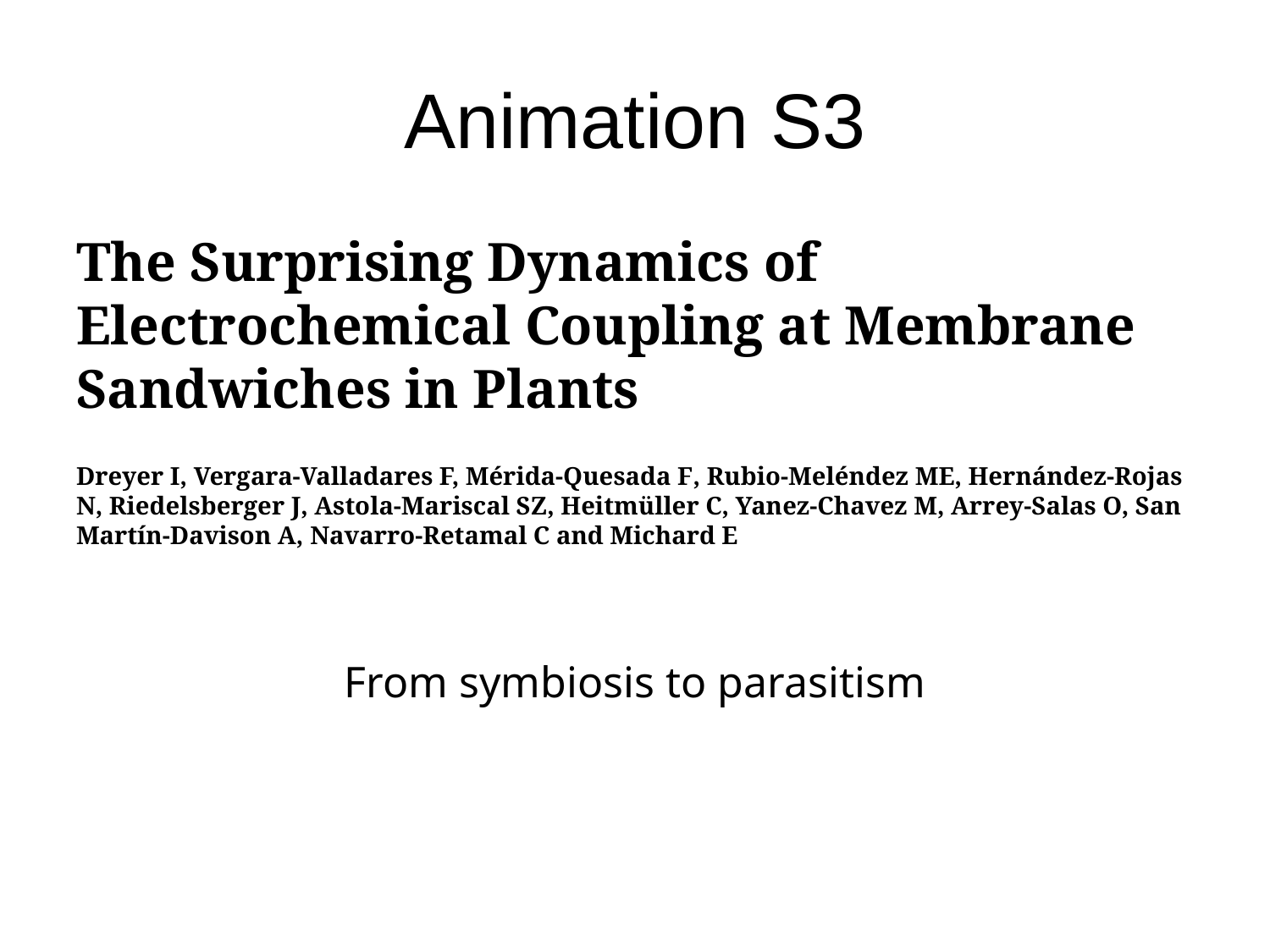

# Animation S3
The Surprising Dynamics of Electrochemical Coupling at Membrane Sandwiches in Plants
Dreyer I, Vergara-Valladares F, Mérida-Quesada F, Rubio-Meléndez ME, Hernández-Rojas N, Riedelsberger J, Astola-Mariscal SZ, Heitmüller C, Yanez-Chavez M, Arrey-Salas O, San Martín-Davison A, Navarro-Retamal C and Michard E
From symbiosis to parasitism

## Slide 2
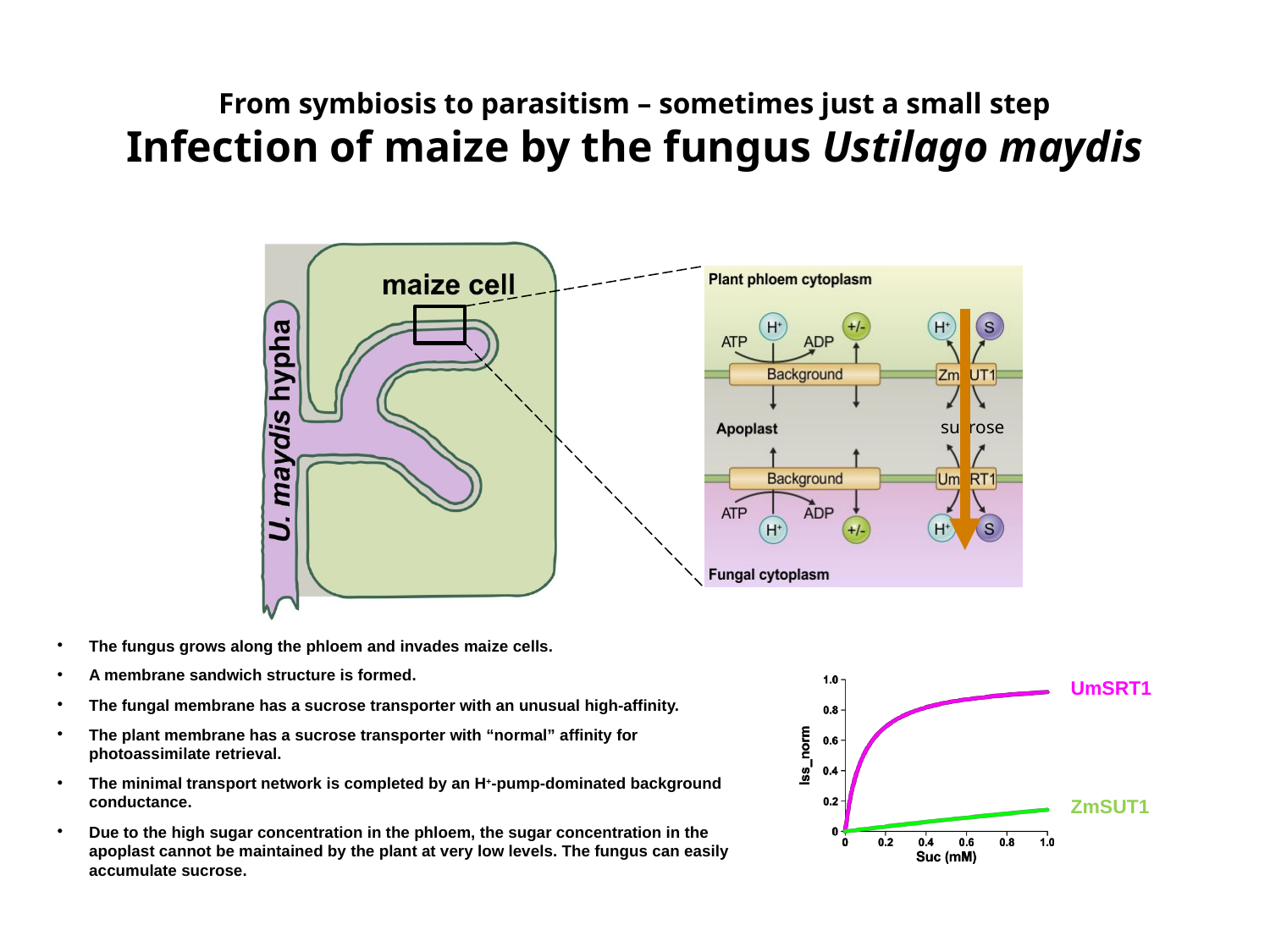

From symbiosis to parasitism – sometimes just a small step
Infection of maize by the fungus Ustilago maydis
sucrose
The fungus grows along the phloem and invades maize cells.
A membrane sandwich structure is formed.
The fungal membrane has a sucrose transporter with an unusual high-affinity.
The plant membrane has a sucrose transporter with “normal” affinity for photoassimilate retrieval.
The minimal transport network is completed by an H+-pump-dominated background conductance.
Due to the high sugar concentration in the phloem, the sugar concentration in the apoplast cannot be maintained by the plant at very low levels. The fungus can easily accumulate sucrose.
UmSRT1
ZmSUT1

## Slide 3
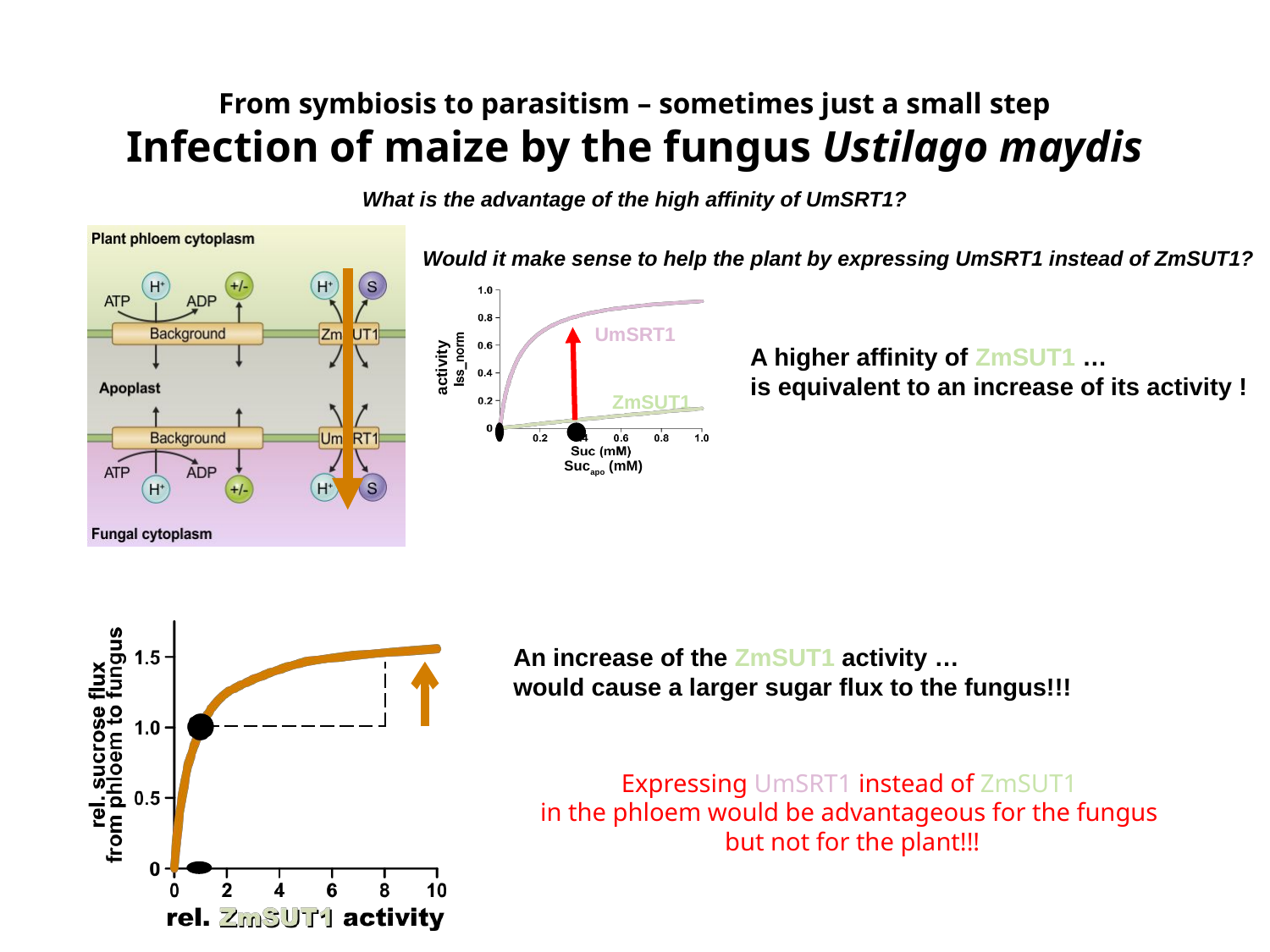

From symbiosis to parasitism – sometimes just a small step
Infection of maize by the fungus Ustilago maydis
What is the advantage of the high affinity of UmSRT1?
Would it make sense to help the plant by expressing UmSRT1 instead of ZmSUT1?
UmSRT1
ZmSUT1
Sucapo (mM)
activity
A higher affinity of ZmSUT1 …
is equivalent to an increase of its activity !
An increase of the ZmSUT1 activity …
would cause a larger sugar flux to the fungus!!!
Expressing UmSRT1 instead of ZmSUT1
in the phloem would be advantageous for the fungus
but not for the plant!!!

## Slide 4
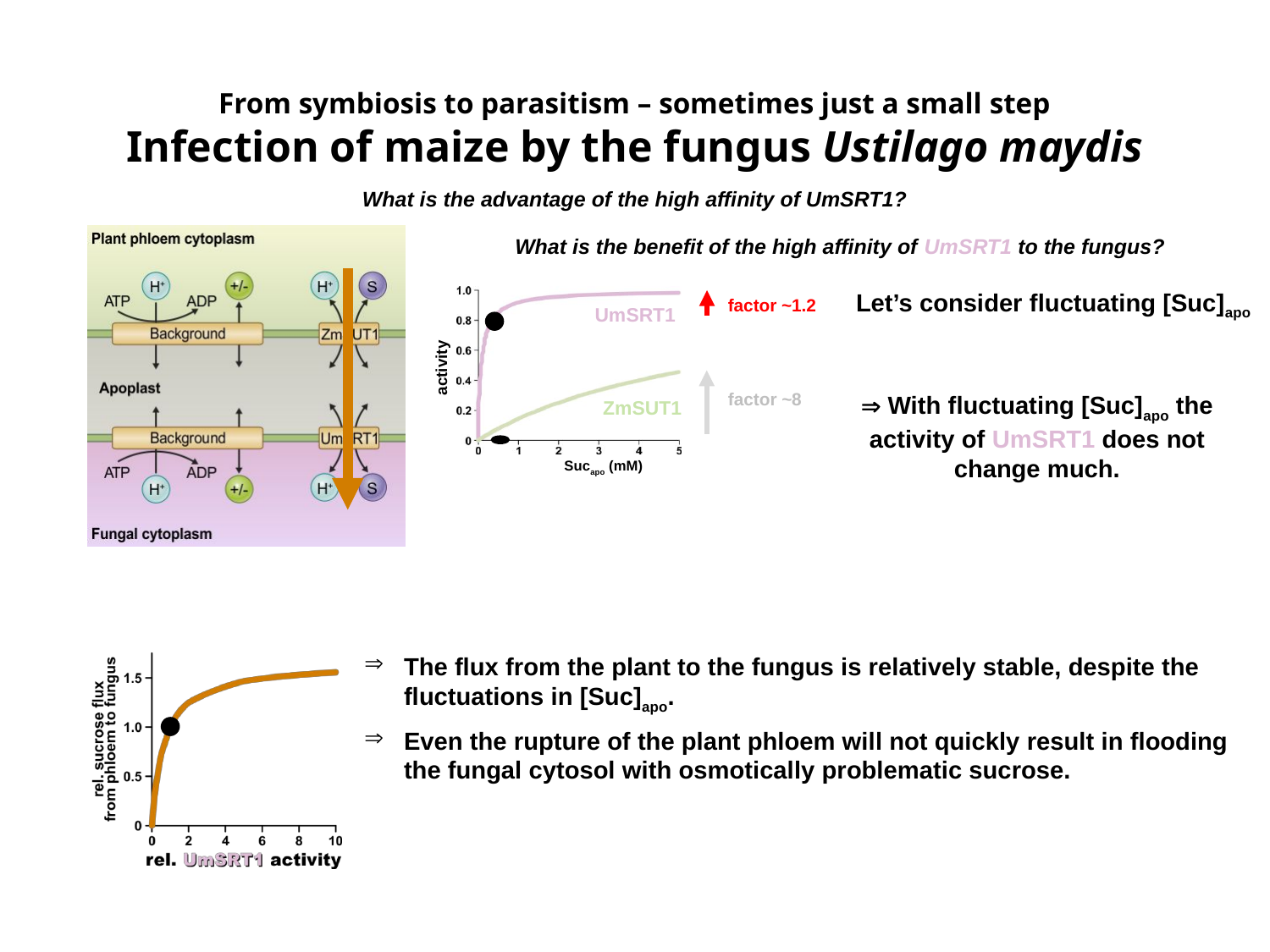

From symbiosis to parasitism – sometimes just a small step
Infection of maize by the fungus Ustilago maydis
What is the advantage of the high affinity of UmSRT1?
What is the benefit of the high affinity of UmSRT1 to the fungus?
Let’s consider fluctuating [Suc]apo
UmSRT1
activity
ZmSUT1
Sucapo (mM)
factor ~1.2
factor ~8
 With fluctuating [Suc]apo the activity of UmSRT1 does not change much.
The flux from the plant to the fungus is relatively stable, despite the fluctuations in [Suc]apo.
Even the rupture of the plant phloem will not quickly result in flooding the fungal cytosol with osmotically problematic sucrose.

## Slide 5
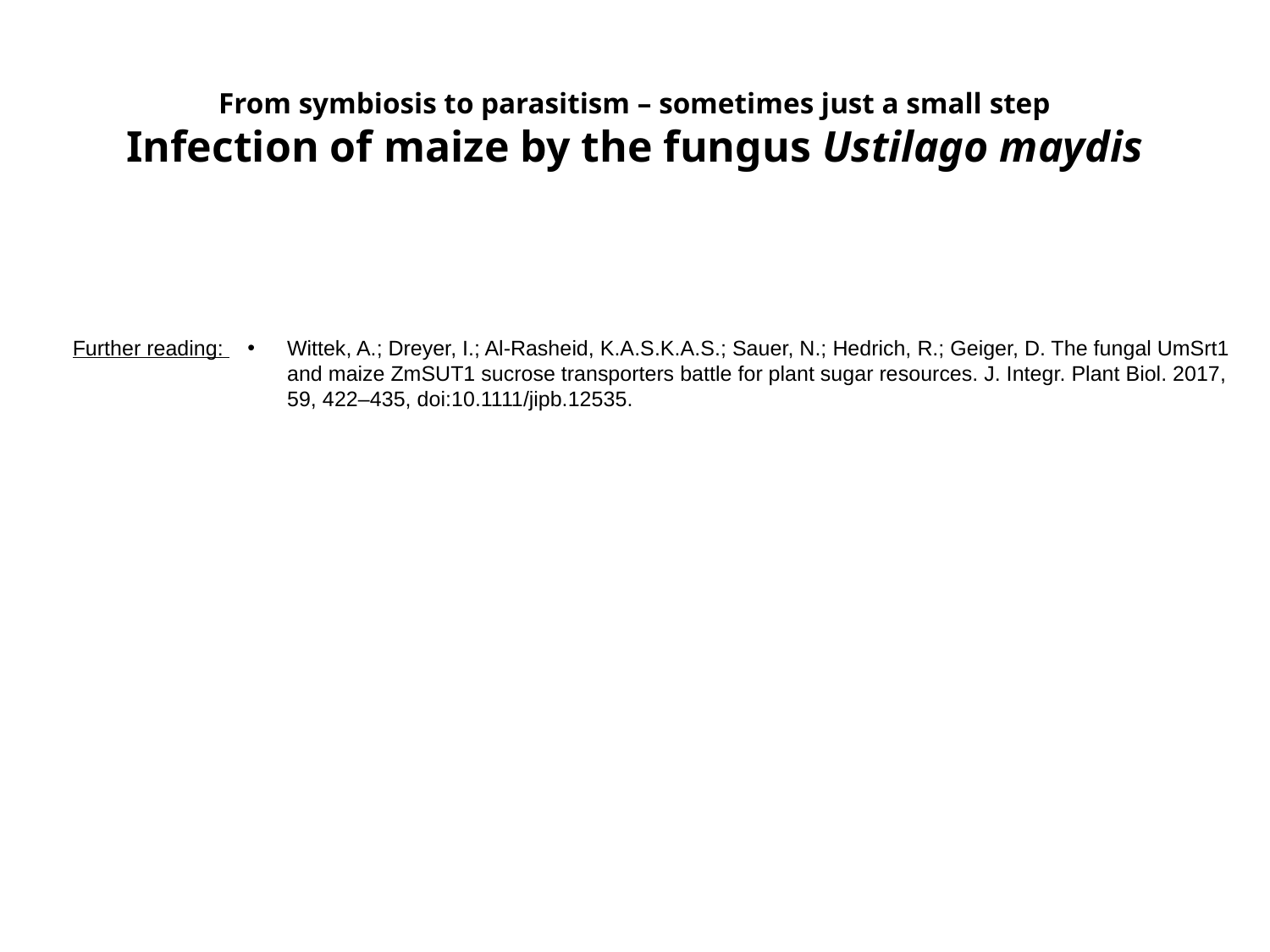

From symbiosis to parasitism – sometimes just a small step
Infection of maize by the fungus Ustilago maydis
Further reading:
Wittek, A.; Dreyer, I.; Al-Rasheid, K.A.S.K.A.S.; Sauer, N.; Hedrich, R.; Geiger, D. The fungal UmSrt1 and maize ZmSUT1 sucrose transporters battle for plant sugar resources. J. Integr. Plant Biol. 2017, 59, 422–435, doi:10.1111/jipb.12535.
